# Letter-Sound Knowledge: Exploring Gender Differences in Children When They Start School Regarding Knowledge of Large Letters, Small Letters, Sound Large Letters, and Sound Small Letters

**DOI:** 10.3389/fpsyg.2017.01539

**Published:** 2017-09-08

**Authors:** Hermundur Sigmundsson, Adrian D. Eriksen, Greta Storm Ofteland, Monika Haga

**Affiliations:** ^1^Department of Psychology, Norwegian University of Science and Technology Trondheim, Norway; ^2^Reykjavik University Reykjavik, Iceland; ^3^Department of Neuromedicine and Movement Science, Norwegian University of Science and Technology Trondheim, Norway

**Keywords:** children, gender differences, letter-sound knowledge

## Abstract

This study explored whether there is a gender difference in letter-sound knowledge when children start at school. 485 children aged 5–6 years completed assessment of letter-sound knowledge, i.e., large letters; sound of large letters; small letters; sound of small letters. The findings indicate a significant difference between girls and boys in all four factors tested in this study in favor of the girls. There are still no clear explanations to the basis of a presumed gender difference in letter-sound knowledge. That the findings have origin in neuro-biological factors cannot be excluded, however, the fact that girls probably have been exposed to more language experience/stimulation compared to boys, lends support to explanations derived from environmental aspects.

## Introduction

The four most prominent communication skills to children’s intellectual, emotional and social developments are speaking, listening, reading, and writing (Rose, 2006). Students failing to acquire basic reading skills in early grades have greater risks of academic shortcomings and problematic behavior later on Adams (1990), Elbaum et al. (2000), and Tønnessen and Uppstad (2015). Large scale academic assessments such as PISA and PIRLS have shown a large gender gap in reading (Mullis et al., 2012; Stoet and Geary, 2013; OECD, 2014).

Phonological awareness is considered as an important enabling skill in reading and writing. In general, there has been little attention to gender differences in phonological awareness, however, studies have found that girls perform superior compared to boys at the age of 6 years (Lundberg et al., 2012).

Girls tend to have better achievements in reading (Stoet and Geary, 2013). More specifically, gender differences in vocabulary growth (Huttenlocher et al., 1991), letter writing scores (Puranik et al., 2013) and word recognition (Samuels and Turnure, 1974) have been found among children in preschool and first grade. These differences might have an accumulating effect in academic achievement throughout elementary school (Mullis et al., 2012; cf. OECD, 2014). Girls at age 15 achieve better results than boys in reading, according to an analysis of 10 years of PISA-testing and the gender differences seems to be persistent and growing (Stoet and Geary, 2013).

Research has shown that learning the core principles of the connection between symbols and sounds are essential for the development of reading (Adams, 1990; Rose, 2006; Dehaene and Cohen, 2011). The synthetic phonics method (i.e., focus on letter-sound knowledge) seems to be of critical importance when children are learning to read (Rayner et al., 2001; Rose, 2006; Dehaene and Cohen, 2011; Yoncheva et al., 2015). The alphabetic principle is essentially connecting a letter to a sound, a grapheme to a phoneme, and this approach seems to be more fruitful, especially for individuals with learning disorders such as developmental dyslexia (Rayner et al., 2001). In addition, letter name knowledge is recognized as an important predictor of reading readiness (Chall, 1967; Snow et al., 1998). Knowledge of letters and sound, rather than word-sound knowledge, has a bigger impact on reading achievement (Schneider et al., 2000; Levin et al., 2006; Dehaene, 2011) and makes for a stronger predictor than other predictors combined, such as the child’s tested IQ and cognitive functioning (Chall, 1967; Scanlon and Vellutino, 1996).

At the biological level, neuroimaging studies have found that the areas processing letters and corresponding sounds are very specific, down to details such as orientations of lines (see for example Dehaene and Cohen, 2011). The normative neuro-cognitive area of letter recognition is hypothesized to take place in the left lateral occipitotemporal sulcus (Dehaene et al., 2002; Dehaene and Cohen, 2011). Dehaene et al. (2002) found that this area, aptly named the visual word form area (VWFA), processes words before semantics, and before phonology is attached to the initial symbol. In essence, this means that we are recycling areas in the brain that have evolved for recognizing certain symbols, shapes and faces. Damages or abnormalities to the VWFA have been known to cause reading disabilities and even apraxia (Dehaene and Cohen, 2011). At a neurological level, it is suggested that boys are somewhat slower to develop integration of phonological and visual information compared to girls (Johnston et al., 2012). Other underlying factors such as sex hormones (Giedd et al., 2012), processing speed (Dekker et al., 2013) and visuospatial working memory (Stoet and Geary, 2013) might also account for gender differences in reading. Further evidence of gender differences suggests that girls have deeper engagement and motivation for reading early in development and that this might come from a matter of interest (McKenna et al., 1995; Lynn and Mikk, 2009). On average boys prefer other activities than reading such as computer games or physical activity. This also implies that girls have more reading experience when starting school.

In this respect it is important to be aware of the girls also tend to be more people oriented and boys more thing-oriented, which may affect the early development of language (Halpern, 2012).

Based on evidence from large scale assertions and experimental studies, one essential factor seems to be specificity in reading instruction (Rayner et al., 2001; Levin et al., 2006; Rose, 2006; Dehaene and Cohen, 2011; Tønnessen and Uppstad, 2015). Poor readers tend to benefit more from the synthetic phonics method (Rose, 2006). Current evidence shows that biological as well as behavioral gender differences call for specialized reading instruction.

Based on previous studies focusing on literacy (Samuels and Turnure, 1974; Huttenlocher et al., 1991; Puranik et al., 2013) we would expect some gender differences in letter-sound knowledge in early childhood. However, research on gender differences in this topic are both contradicting and scarse (Dodd and Carr, 2003). As a consequence there is little knowledge about when these possible gender differences emerge during childhood.

The aim of this study was to examine gender differences in letter sound knowledge when children start at school at age 5–6 years. The possible findings of gender differences in letter-sound knowledge when children start at school may be important because it could influence our teaching approaches.

## Materials and Methods

### Study Design and Participants

Total of 485 children aged between 5 and 6 years were recruited for this study. The participants completed an assessment of letter-sound knowledge (Bokstavtesten) (Ofteland, 1992).

The children *N* = 485, 224 girls and 261 boys, were recruited from 28 schools in county in Norway (convenience sampling). The mean chronological age for the entire group was 6,14 (*SD* = 0.28) years; the overall range was 5,67 to 6,67 years.

The entire sample reflected the population of children attending schools in these areas and included children in a wide range of socio-economic backgrounds. No child had any behavioral, neurological or orthopedic problem or any reported history of learning difficulties that would qualify as exclusions criteria for this study. All the participants had no primary uncorrected visual deficit; no medical condition that might interfere with their ability to carry out the tests.

### Measurements

#### Letter-Sound Knowledge

Letter-sound knowledge was assessed with the Bokstavtesten [Letter-sound knowledge test (LSK test), Norwegian version] (Ofteland, 1992).

In the LSK, the participants should do following:

1. Indicate how many of the large letters of alphabet they know (ABC…)
2. Indicate how many of the large letters of alphabet they know the sound to
3. Indicate how many of the small letters of the alphabet they know (a,b,c….)
4. Indicate how many of the small letters of the alphabet they know the sound to

In Norway there are 29 letters in the Alphabet.

The tests take around 10 min per participant. The LSK test has two sheets, one for the large letters and other for the small letters.

The test has proved to be a reliable and valid test of isolated word decoding proficiency (Ofteland, 1992). We estimated the convergent construct validity of the test battery by comparing the rankings of 20 Norwegian children 6 years old (mean age: 6.05, *SD*: 0.28) in one class based on test scores, with the rankings of the same children on the basis of an evaluation of their teacher. There was a close association between the rankings based on the teacher’s evaluation and the ranking of test scores. This association was confirmed by Spearman rho correlations between the two rank scores, which were 0.683.

We estimated the relative test–retest reliability of the test-battery by using ICC (2,1) (Shrout and Fleiss, 1979). The results indicated good reliability for individual test item scores, with ICCs between test and retest scores ranging from 0.985 to 0.992 for 6-years old (mean age: 6.05, *SD*: 0.28) Norwegian children (*N* = 20).

### Procedure

Full ethical review and approval was not required for this study in accordance with the national and institutional guidelines, however, the study was carried out in accordance with the recommendations of Norwegian Centre for Research Data and the Declaration of Helsinki. Written informed consent was obtained from the parents of all participants prior to the study commencement. Identification numbers were used to maintain data confidentiality.

The assessment took place in a quiet room during normal school hours and was conducted in accordance with the LSK manual. Children were tested at the start of their first school year in August/September. All the participants were tested individually by their teachers that had been trained in the test protocols. Each test item was explained and demonstrated before the participants started. Participants were given verbal encouragement and support throughout the testing procedure. If the participants made a procedural error, instructions and demonstrations were repeated and the participant made a new attempt.

### Data Reduction and Analysis

For the statistical analysis, SPSS Version 19 for Windows was used (SPSS, Inc., Chicago, IL, United States). Descriptive statistics was used to calculate the mean and standard deviation of score for number of letters. MANOVA were used for between group analyses for gender.

Higher scores indicate higher performance on the tasks. Statistical significance was set at *p* < 0.05.

## Results

**FIGURE 1:**
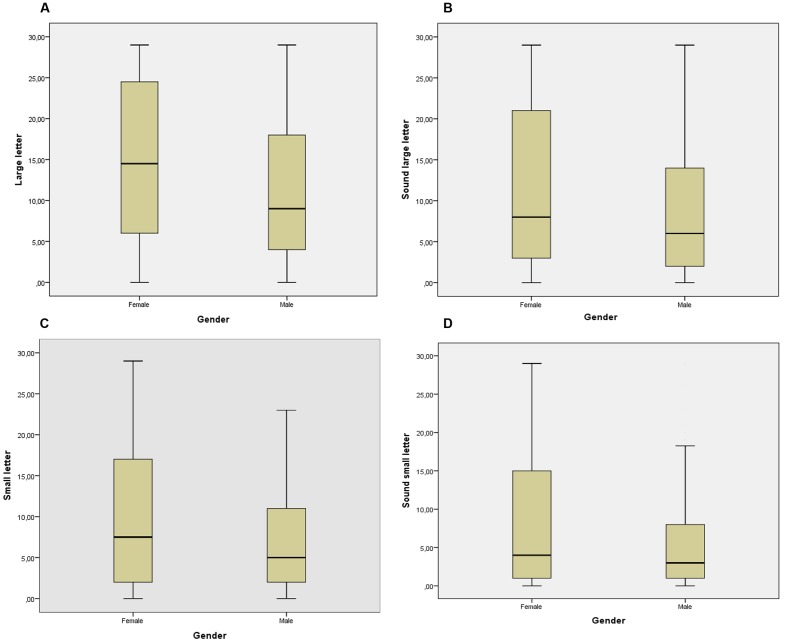
Box plots depicting the female and male performance on letter-sound knowledge test. **(A)** Large letter; **(B)** sound large letter; **(C)** small letter; **(D)** sound small letters. Horizontal lines within boxes represent the group medians. Box edges define the first and third quartiles, whiskers define the 10 and 90%.

Descriptive statistics of score for number of large letters, sound large letters, small letters, and sound of small letters for the 5–6 year old girls and boys are shown in **Figures 1A–D**. Higher scores indicate better performance (better knowledge of letters and sound).

MANOVA indicate significant main effect of gender [*F*(1,485 = 11.816, *p* < 0.001], with a low effect size ($\eta_{p}^{2}$ = 0.028). Thus, there was an overall difference between girls and boys.

### Letter Large

Mean (SD): girls 14.83 (9.91); boys 11.51 (8.81). A significant mean effect was obtained for gender [*F*(1,485) = 11.925, *p* < 0.001 (MANOVA), with a low effect size ($\eta_{p}^{2}$ = 0.028)] (**Figure 1A**).

### Sound Letter Large

Mean (SD): girls 11.63 (9.89); boys 8.67 (8.45). A significant mean effect was obtained for gender [*F*(1,485) = 8.285, *p* < 0.004 (MANOVA), with a low effect size ($\eta_{p}^{2}$ = 0.020)] (**Figure 1B**).

### Letter Small

Mean (SD): girls 10.35 (9.53); boys 7.31 (7.51). A significant mean effect was obtained for gender [*F*(1,485) = 13.072, *p* < 0.001 (MANOVA), with a low effect size ($\eta_{p}^{2}$ = 0.031)] (**Figure 1C**).

### Sound Letter Small

Mean (SD): girls 8.62 (9.63); boys 5.86 (7.42). A significant mean effect was obtained for gender [*F*(1,485) = 10.815, *p* < 0.001 (MANOVA), with a low effect size ($\eta_{p}^{2}$ = 0.026)] (**Figure 1D**).

#### Correlation between the Four Factors

Girls: Large letter – sound large letter: *r* = 0.897; Large letter – small letter: *r* = 0.929; Large letter – sound small letter: *r* = 0.848; Sound large letter – small letter: *r* = 0.929; Sound large letter – sound small letter: *r* = 0.941; Small letter – sound small letter: *r* = 0.939 (Pearson correlations).

Boys: Large letter – sound large letter: *r* = 0.905; Large letter – small letter: *r* = 0.914; Large letter – sound small letter: *r* = 0.854; Sound large letter – small letter: *r* = 0.862; Sound large letter – sound small letter: *r* = 0.918; Small letter – sound small letter: *r* = 0.938 (Pearson correlations).

The correlations are high and significant (*p* < 0.001) and similar between the genders.

## Discussion

The aim of this study was to examine gender differences in letter sound knowledge when children start at school at age 5–6 years.

Analyses show a high correlation between the four factors for both girls and boys. The findings indicate a significant difference between girls and boys in all four factors tested in this study, i.e., number of large letters, sound large letter, small letters and sound small letters, in favor of the girls. This indicates an early emerging gap between the genders in letter-sound knowledge, in accordance with findings from previous research (Mullis et al., 2003, 2007, 2012; cf. OECD, 2014). Letter-sound knowledge is one of the most important factors for reading development (Schneider et al., 2000; Levin et al., 2006; Dehaene, 2011) and is a stronger predictor for reading achievement than a child’s cognitive functioning and intelligence measurements (Chall, 1967; Scanlon and Vellutino, 1996).

Dehaene (2011) argues that: ‘Grapheme-phoneme correspondences must be systematically taught, one by one: the amount of such teaching is the best predictor of reading performance…’ (p. 26). This was earlier pointed out by Ehri et al. (2001) who in their meta-analysis found that systematic phonics instruction facilitated children to read more effectively than non-systematic or no phonics instruction (p. 417). Rose (2006) argues that boys seem to benefit the most from systematic phonics instruction. The finding of differences in letter-sound knowledge before children start at school is important knowledge because it could influence the way we organize and teach at an early stage in order to minimize the gap- and not amplifying it through schooling. As the letter-sound knowledge is a strong predictor of reading development it would also be important for academic performance in general.

Earlier research indicates that at age 14 there are extensive gender differences in reading skills (OECD, 2016). In Norway 21% of adolescent boys and 9% of girls do not read so well that they are able to understand the text they are reading. In Iceland, the number is even higher, 28% of the boys and 15% of the girls respectively (Kjærnsli and Jensen, 2016). Further, international studies on reading comprehension with 10 year-old children found gender differences in favor of the girls for every participating country (i.e., 35 to 40 countries) (Mullis et al., 2003, 2007).

The explanation of the gap may be related to both nature and nurture ‘multicausal’ (Stoet and Geary, 2013). There is consistent evidence that gender differences, in favor of girls, exists in vocabulary growth among children below 2 years of age (Reznick and Goldfield, 1989). In this respect Hohm et al. (2007) found a significant difference between genders on both expressive and receptive language (age-equivalent scores), in favor of girls, at the age of 10 months.

This may indicate a nature explanation, i.e., that there are maturational differences in the language capacities of girls and boys (Huttenlocher et al., 1991; McCune, 1992). However, focusing only on this nature explanation might not give the right picture because mothers tend to talk more with girls than the boys, i.e., nurture explanation (Halverson and Waldrop, 1970; Cherry and Lewis, 1978). These gender differences in vocabulary growth are only found until 2 years of age but not after that age (Huttenlocher et al., 1991). This lends support to Gottlieb’s (1998) probabilistic epigenesis theory suggesting that individual development is always an interaction between genes, neural system, behavior and environment, i.e., experience and learning, it is hard to say whether the reasons for the gender gap are a function of biology, school practices or cultural influences (OECD, 2015).

The finding of gender differences in the age group 5–6 year may also be explained by earlier maturation in neural network of importance to executive functions in girls than boys (Giedd et al., 2012; Dekker et al., 2013; Stoet and Geary, 2013). More efficient executive functions may have influences on for example concentration, which is a key factor for learning. This may also be related to the findings that girls perform superior compared to boys at the age of 6 years in phonological awareness (Lundberg et al., 2012) as girls may have been exposed to more language experience/stimulation compared to boys.

Findings also indicate that girls have more favorable attitudes toward reading and reading motivation than boys (McKenna et al., 1995; Lynn and Mikk, 2009).

The findings from this study may suggest that it is of relevance to test letter-sound knowledge at an early stage in childhood as when they attend school. Furthermore, early intervention/stimulation in children with a low performance in letter-sound knowledge may be advantageous. Research indicates that to develop skills, such as reading, specific training is needed (Kleim and Jones, 2008). Dehaene et al. (2010) argues that learning to read means reorganizing neural circuits in the brain. In other words, the changes that reading makes to our brain results from the effect of our experience with reading. These findings can be used as an argument for the task specificity principles of learning across cognitive and motor skills, as the processes associated with learning may seem relatively independent and specific (Kleim and Jones, 2008; Sigmundsson et al., 2013, 2017; Stöckel and Hughes, 2015). Gender differences in reading is both growing and consistent (Stoet and Geary, 2013) yet it is ignored in the literature (Dodd and Carr, 2003). This implies the need for further exploration on the multicausal nature of gender differences in reading and language skills especially in preschool and school start.

## Author Contributions

HS: idea, analysis, writing; AE: analysis, writing; GO: idea, writing; MH: idea, analysis, writing.

## Conflict of Interest Statement

The authors declare that the research was conducted in the absence of any commercial or financial relationships that could be construed as a potential conflict of interest.
